# Comparative effects of RRR-alpha- and RRR-gamma-tocopherol on proliferation and apoptosis in human colon cancer cell lines

**DOI:** 10.1186/1471-2407-6-13

**Published:** 2006-01-17

**Authors:** Sharon E Campbell, William L Stone, Steven Lee, Sarah Whaley, Hongsong Yang, Min Qui, Paige Goforth, Devin Sherman, Derek McHaffie, Koyamangalath Krishnan

**Affiliations:** 1 Department of Internal Medicine, James H. Quillen College of Medicine, East Tennessee State University, Johnson City, TN 37614, USA; 2 Department of Pediatrics, James H. Quillen College of Medicine, East Tennessee State University, Johnson City, TN 37614, USA; 3 Department of Clinical Cancer Prevention and Gastrointestinal Medical Oncology, The University of Texas MD Anderson Cancer Center, Houston, TX 770303, USA

## Abstract

**Background:**

Mediterranean societies, with diets rich in vitamin E isoforms, have a lower risk for colon cancer than those of northern Europe and the Americas. Vitamin E rich diets may neutralize free radicals generated by fecal bacteria in the gut and prevent DNA damage, but signal transduction activities can occur independent of the antioxidant function. The term vitamin E represents eight structurally related compounds, each differing in their potency and mechanisms of chemoprevention. The RRR-γ-tocopherol isoform is found primarily in the US diet, while RRR-α-tocopherol is highest in the plasma.

**Methods:**

The effectiveness of RRR-α- and RRR-γ-tocopherol at inhibiting cell growth and inducing apoptosis in colon cancer cell lines with varying molecular characteristics (SW480, HCT-15, HCT-116 and HT-29) and primary colon cells (CCD-112CoN, nontransformed normal phenotype) was studied. Colon cells were treated with and without RRR-α- or RRR-γ-tocopherol using varying tocopherol concentrations and time intervals. Cell proliferation and apoptosis were measured using the trypan blue assay, annexin V staining, DNA laddering and caspase activation.

**Results:**

Treatment with RRR-γ-tocopherol resulted in significant cell death for all cancer cell lines tested, while RRR-α-tocopherol did not. Further, RRR-γ-tocopherol treatment showed no cytotoxicity to normal colon cells CCD-112CoN at the highest concentration and time point tested. RRR-γ-tocopherol treatment resulted in cleavage of PARP, caspase 3, 7, and 8, but not caspase 9. Differences in the percentage cell death and apoptosis were observed in different cell lines suggesting that molecular differences in these cell lines may influence the ability of RRR-γ-tocopherol to induce cell death.

**Conclusion:**

This is the first study to demonstrate that multiple colon cancer cell lines containing varying genetic alterations will under go growth reduction and apoptosis in the presence of RRR-γ-tocopherol without damage to normal colon cells. The amount growth reduction was dependent upon the molecular signatures of the cell lines. Since RRR-γ-tocopherol is effective at inhibition of cell proliferation at both physiological and pharmacological concentrations dietary RRR-γ-tocopherol may be chemopreventive, while pharmacological concentrations of RRR-γ-tocopherol may aid chemotherapy without toxic effects to normal cells demonstrated by most chemotherapeutic agents.

## Background

Differences in the incidence of colorectal cancer throughout the world suggest that diet has a role in colorectal carcinogenesis [1]. Mediterranean diets, rich in vitamin E isoforms, are associated with a lower incidence of colon cancer [2,3]. Vitamin E refers to any of four tocopherols or tocotrienols (α, β, δ, and γ) isoforms. All of the tocopherol isoforms found in nature are the RRR-forms since all three chiral carbons on the side chain attached to the chroman head group have the "R-" configuration rather then the "S-" configuration. Synthetic vitamin E (typically found in dietary supplements) almost always refers to all-rac-alpha-tocopherol which is a racemic mixture containing eight stereo isomers, one eighth of which is the biologically active RRR-isoform. The isoform found in highest concentration in the serum is the RRR-α-tocopherol. The primary form of vitamin E found in the North American diet is RRR-γ-tocopherol, which is present at levels 2–4 times higher than that of RRR-α-tocopherol [4].

While most clinical studies with vitamin E have used all-rac-α-tocopherol, recent studies show that RRR-γ-tocopherol also may play a unique role in preventing colon cancer [5]. Epidemiological evidence shows that RRR-γ-tocopherol levels in plasma correlate with a reduced risk for both colon and prostate cancer [6-8]. Animal studies measuring direct end-points of cancer (i.e., survival studies, tumor reduction, tumor prevention) with RRR-γ-tocopherol are lacking. Vitamin E was demonstrated a chemopreventive in animal models of chemically induced colon cancer [9], but these studies tested only α-tocopherol or α-tocopherol acetate (RRR- or all-rac- not specified). Animal studies measuring the potential chemopreventive mechanisms of RRR-γ-tocopherol have used surrogate biomarkers of carcinogenesis. For example, RRR-γ-tocopherol can suppress the expression of ras p-21 in rat colonocytes *in vivo *[10]. Using Wistar rats, Jiang has demonstrated that RRR-γ-tocopherol, but not α-tocopherol (RRR- or all-rac- not specified) decreases the proinflammatory eicosanoid PGE_2 _(which is known to play a role in the progression of colorectal cancer through inflammation). Further, hemodialysis patients administered RRR-γ-enriched tocopherols showed a consistently lower level of C-reactive protein (a biomarker of inflammation) while the administration of α-enriched tocopherols (RRR- or all-rac- not specified) did not [11].

Cooney *et al. *found that RRR-γ-tocopherol was a much more potent inhibitor of neoplastic transformation in 3-methycholanthrene-treated C3H/H10T/1/2 murine fibroblasts than α-tocopherol (RRR- or all-rac- not specified) [12]. In addition, RRR-γ-tocopherol is a potent inhibitor of COX-2 activity and inhibits human cancer cell cycle progression and cell proliferation by down-regulation of cyclins [13,14]. Stone *et al. *have found that RRR-γ-tocopherol is taken up by RAW 264.7 macrophages to a much greater extent than RRR-α-tocopherol [15].

Vitamin E isoforms (and metabolites) have had varying effectiveness at inhibiting cell growth and inducing apoptosis. RRR-γ-Tocopherol is superior to all-rac-α-tocopherol at cell growth inhibition *in vitro *in human prostate cancer cells [16]. Vitamin E succinate (VES) and tocotrienols demonstrate potent apoptotic inducing properties [17]. Zu *et al. *[18] have shown differential synergistic effects of selenium with vitamin E isoforms on cell growth and apoptosis in PC-3 human prostate cancer cells. VES was the most effective form tested and synergizes with selenium, while RRR-α-tocopherol and RRR-α-tocopheryl acetate were weaker in their effects on suppressing growth and inducing apoptosis in PC-3 prostate cancer cells. α-Tocopherol (RRR or all-rac not specified) is a poor inducer of apoptosis in the colon cancer xenograft nude mouse model, while the synthetic form, α-tocopheryl succinate (a redox inactive analogue of vitamin E) is a strong inducer of apoptosis [19]. In addition, carboxyethyl hydroxychromans (CEHC) metabolites of γ-tocopherol are powerful inducers of apoptosis and inhibit cell growth and down regulate cyclin expression in PC-3 prostate cancer cell lines [20]. The performance of vitamin E derivatives and analogues with regard to apoptosis also vary among tissue types. VES and a vitamin E analogue, 2,5,7,8-tetramethyl-2R-(4R,8R,12-trimethyltridecyl)chroman-6-yloxy acetic acid (α-TEA) can induce human breast, prostate, colon, lung, cervical, and endometrial tumor cells, but the apoptotic-inducing effects of α-TEA are greater in human ovarian and cervical cancer cells [21]. To date, no study has compared the anti-proliferative and apoptotic effects of α- and γ-tocopherol on colon cancer cell lines with differences in molecular features. Gysin *et al. *demonstrated the inhibition of cell proliferation by RRR-γ-tocopherol treatment at 25 μM on CaCo-2 (colon carcinoma), DU-145 and LNCap (prostate carcinoma) and SaOs-2 (osteosarcoma) cells, but demonstrated no apoptosis in any of the cell lines tested.

In this study, we compared the anti-proliferative and apoptotic effects of RRR-α-tocopherol and RRR-γ-tocopherol in four colon cancer cell lines with varying molecular characteristics, SW480 (APC, type I truncation and COX-2 deficient), HCT-15 (COX-2 deficient), HCT-116 (APC, wild-type and COX-2 inducible), and HT-29 (APC, type II truncation and COX-2 constitutive expression) and normal untransformed colon cells (CCD-12CoN). Proliferation studies demonstrated that RRR-γ-tocopherol is able to inhibit proliferation and cause cell death in all four cancer cell lines, but not in the normal cells. In the colon cancer cell lines tested for apoptosis, we have shown that RRR-γ-tocopherol activates cleavage of PARP and caspase 3, 7 and 8, but not caspase 9. Dietary RRR-γ-tocopherol may be an effective colon cancer preventive agent while pharmacological concentrations of RRR-γ-tocopherol may be useful as an adjuvant chemotherapy agent resulting lower dose of agents that are cytotoxic to normal cells.

## Methods

### Chemicals

α-Tocopherol (Eastman Chemical, Kingsport, TN, 99% pure RRR-α-tocopherol), γ-tocopherol (Tama Biochemical, Tokyo, Japan, 97% pure RRR-γ-tocopherol), troglitazone (BioMol Research Lab, Plymouth Meeting, PA), 15-deoxyΔ12,14-PGJ_2 _(BioMol Research Lab, Plymouth Meeting, PA), bovine serum albumin (Gibco BRL, Gaithersburg, MD), proteinase K (Sigma Chemical, St. Louis, MO), RNAse A (Sigma Chemical, St. Louis, MO), and camptothecin (Sigma Chemical, St. Louis, MO) were obtained from the indicated sources.

### Cell culture

The colon cancer cell lines SW480 (RPMI 1640), HCT-116 (McCoy's), HT-29 (Dulbecco's Modified Eagle Medium), and HCT-15 (RPMI 1640) were purchased from American Type Culture Collection, ATCC (Manassas, VA) and grown in the indicated media supplemented with 10% FBS and 50 IU penicillin/streptomycin in a humidified atmosphere of 5% CO_2 _at 37°C. The CCD-112CoN (normal primary cells, nontransformed phenotype) were grown in a humidified atmosphere of 5% CO2 at 37°C with Dulbecco's Modified Eagle's and supplemented with 10% FBS.

### Enrichment of tocopherol into tissue culture medium

Prior to treatment, the cell culture medium was enriched with tocopherol by adding the appropriate amount of tocopherol in ethanol, followed by five volumes of bovine serum albumin (BSA). The BSA/tocopherol mixture was vortexed and added to appropriate culture medium. In the vehicle-treated cells, the tocopherol was omitted from the BSA/ethanol mixture which was added to the appropriate culture medium supplemented with 10% FBS.

### Percent dead assay

Percent cell death was measured by using the Live-Dead Assay according to the manufacturer's instructions (Molecular Probes, Eugene, OR) with a Gemini XS fluorimeter (Molecular Devices, Sunnyvale, CA). The percentage of cell death was calculated by measuring the fluorescence intensity of the ethidium bromide homodimer (EthD-1) that is excluded from live cells, but enters the cells with damaged membranes and undergoes a fluorescence enhancement upon binding to nucleic acids in dead cells (λ_ex _= 530 nm and λ_em _= 645 nm). Control samples for percent dead calculations were verified for viability before and after plating using the trypan blue exclusion assay. EthD-1 dye and cell concentrations were optimized according to manufacturers' instructions. SW480 cells were seeded at a density of 5 × 10^4 ^cells/well while HCT-116 cells were plated at a density of 2 × 10^4 ^cells/well in 96-well plates for 24 hours before treatment. Vitamin E-enriched PBS (previously described) was added at 25, 50, 100, 150 and 200 μM concentrations. Cells were treated for five hours.

### Cell viability and cell death analysis

SW480, HT-29, HCT-116 and HCT-15 cells were seeded at 1.5 × 10^5 ^cells/well in 12 well plates 24 hours prior to tocopherol treatment for time intervals to 72 hours. Tocopherol-enriched media was added to a concentration up to 100 μM tocopherol. This concentration was selected after performing Live-Dead Assays (Molecular Probes) and determining that it was the lowest concentration where cell death was mediated by α-tocopherol. We selected this concentration to determine if α-tocopherol could induce apoptosis. Cells were removed from flasks by trypsinization at indicated times and counted with a hemocytometer using trypan blue staining and the Beckman Coulter Z2 cell counter. The cells were assayed by trypan blue exclusion stain as described above. At lower tocopherol concentrations and incubation times the cells were seeded at 1 × 10^4 ^cells/well. Tocopherol enriched media was added to the appropriate concentration at the zero time interval and was replenished every 72 hours. Cell viability was assayed hemocytometer cell counting with trypan blue dye. Troglitazone, 15-deoxy Δ12,14-PGJ_2_, and camptothecin are capable of inducing cell death and apoptosis in colon cancer cells and were used as positive controls for cell death and apoptosis. Cell viability was measured by live cell counts as a function of time and compared to the vehicle, while cell death was measured as the percentage of dead cells at each time point measured.

### Intracellular analysis of vitamin E by HPLC analysis

The tocopherol content of the treated cell lysates was measured using HPLC analysis with highly sensitive electrochemical detection as previously described [22]. The response factors of tocopherols relative to tocol (an internal standard) were determined in triplicate. The concentrations of RRR-α-tocopherol and RRR-γ-tocopherol were measured using a Spectronic Genesys 5 spectrophotometer and published extinction coefficients [23].

### Apoptosis assays

#### Annexin V and propidium iodide (PI) double staining assay

HCT-116 and SW480 cells were plated in 100 × 20 mm plates at cell densities between 3 × 10^6 ^and 5 × 10^6 ^cells/plate. HCT-116 cells and SW480 cells were treated as described in the trypan blue staining assays. After treatment, cells were removed from the plate with trypsin and analyzed for phosphatidylserine externalization by an annexin V and propidium iodide apoptosis kit (Oncogene Research Products, San Diego, CA) according to the manufacturers' instructions using a Becton Dickson FACS Calibur Flow Cytometer (Becton Dickson, San Diego, CA). Apoptotic cells were counted by flow cytometry and the resulting data analyzed using WinMDI freeware.

#### DNA laddering assay

DNA fragmentation was measured by gel electrophoresis by the standard published procedures. In brief, the cells were plated at a density of 5 × 10^6 ^cells/plate and treated with 100 μM tocopherol-enriched, vehicle-enriched media or 10 μM camptothecin (positive control) for 48 hours. A concentration of 1 × 10^6 ^cells/mL were pelleted and resuspended in lysis buffer (50 mM Tris HCl, 10 mM EDTA, 0.5% SDS). The cell lysates were treated with 20 mg/mL proteinase K and incubated at 55°C for 1 hour followed by addition of 0.5 mg/mL RNAse A and heated to 70°C for 5 minutes. DNA was precipitated with isopropanol, mixed with loading dye (10 mM EDTA, pH 8.0, 40 % sucrose, 0.25 % bromophenol blue) and analyzed on a 2% agarose gel containing 0.5 μg/mL ethidium bromide. Gel images were captured using an Alpha Innotech digital camera equipped with a transilluminator and Alpha Ease 5.5 software (Alpha Innotech Corporation, San Leandro, CA).

#### PARP and caspase cleavage by western blot analysis

The protein concentration of the cells lysates was determined by the BCA protein assay (Pierce Biotechnology, Rockford, IL). Total protein was separated by electrophoresis on a 12% SDS polyacrylamide gel and electro transferred onto Hybond-ECL nitrocellulose membrane using an X cell II Mini Cell Blot module (San Diego, CA). Blotted membranes were incubated with the primary antibodies indicated as follows: caspase 3, 7, 8 and 9 (Cell Signaling Technology, Beverly, MA) and probed with horseradish peroxidase conjugated secondary antibody (Cell Signaling Technology, Beverly, MA). The signal was revealed on hyperfilm using the ECL Western blotting detection reagents 1 and 2 (1 mL each) detection system (Amersham Biosciences, Arlington Heights, IL). To control for consistent loading, membranes were probed with the β-actin antibody (Santa Cruz Biotechnology, Santa Cruz, CA) after stripping the blot with Restore solution (Pierce Biotechnology, Rockford, IL) for 30 minutes at 37°C.

### Statistics

Data is displayed as means with error bars representing standard deviation (SD). One-way analysis of variance (ANOVA) followed by Tukey's test was used to compare the means of live cells in the cell proliferation assays and percentage cell death. Probability levels (p-values) of < 0.05 indicate statistical significance. The values obtained from annexin V staining and cell cycle data are measured as a percentage of the 10,000 events in the analysis. The apoptosis in the control cells are subtracted from the treated samples. The percentage values represent an average percentage apoptotic cells over the control (set at 0%) of two independent assays performed in duplicate.

## Results

### Effects of RRR-α- and RRR-γ-tocopherol treatment on cell death in human colon cancer cells

**Figure 1:**
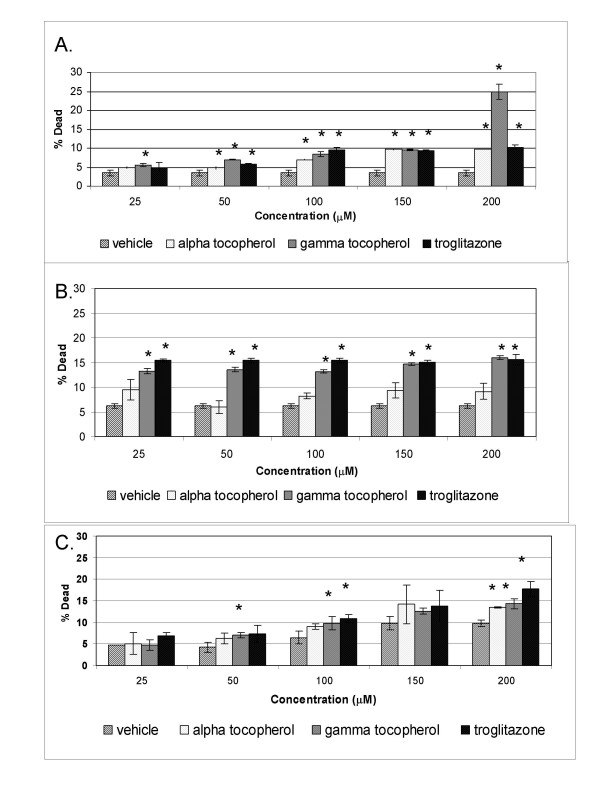
SW480, HCT-116, and HT-29 cells were treated for 5 hours with varying concentrations of tocopherol or the PPAR γ ligand, troglitazone (positive control). The percent of dead cells for SW480 cells (A), HCT-116 cells (B) or HT-29(C) was measured using the Live-Dead assay (Molecular Probes, CA). (Data are representative of three independent trials performed in triplicate. *p < 0.05 vs. vehicle at corresponding concentration.)

Using the Live-Dead Assay (Molecular Probes) cell death was monitored with physiological and pharmacological concentrations of Vitamin E after 5 hours of treatment (Figure 1). The results varied among the cell lines, however RRR-γ-tocopherol demonstrated a concentration-dependent increase in cell death over the vehicle in all cell lines tested. RRR-α-tocopherol did not demonstrate significant cell death over the vehicle at any concentration tested in the HCT-116 and was only significant at 200 μM in the HT-29 cells. The RRR-γ-tocopherol treatment resulted in significant cell death above the vehicle-treated cells in the SW480 and HCT-116 at all concentrations tested (25 μM through 200 μM). For the HT-29 cell line, RRR-γ-tocopherol at levels of 50 μM were effective at inducing cell death. The RRR-α-tocopherol treatment resulted in significant cell death at 100 μM in the SW480 and HCT-116 cell lines, while the HT-29 cells required 200 μM α-tocopherol to increase cell death above the control. One-way ANOVA and tukey's statistical analysis was used to compare the dead cell means for each treatment to that of the vehicle. Asterisks above the bars demonstrate p-values less than 0.05 for cell mean comparison with the vehicle treatment. Post-hoc analysis (Tukey's test) revealed that RRR-γ-tocopherol was significantly better at inducing cell death than RRR-α-tocopherol in SW480 (at all concentrations tested) and HCT-116 cells (at concentrations higher than 50 μM).

Since our goal was to determine if both vitamin E isoforms (RRR-α- and RRR-γ-tocopherol) could induce apoptosis, a concentration was selected whereby cell death could be obtained with both isoforms. When treated with RRR-α-tocopherol, cells did not exhibit cell death at concentrations lower than 100 μM (Figure 1).

### Effects of RRR-α- and RRR-γ-tocopherol treatment on cell proliferation in human colon cancer cells

**Figure 2:**
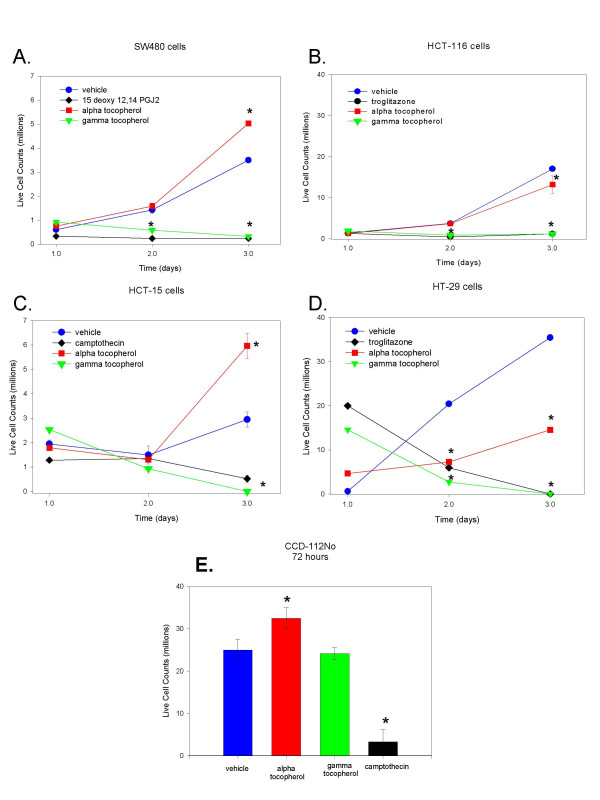
Growth curves for SW480 cells (A) HCT-116 cells (B), HCT-15 cells (C), HT-29 (D) were plotted as average cell counts over time after treatment with 100 μM tocopherol at 1, 2, and 3 days. and Live Cell Counts in CCD-112CoN cells following 100 μM tocopherol treatment at 72 hours (E). Values plotted are averages of three independent trials. Error bars represent standard deviation of the means. Positive controls used included (15 deoxy Δ 12,14 PGJ2, troglitzone and camptothecin). (Data are representative of three independent trials performed in triplicate. *p < 0.05 vs. vehicle at corresponding concentration.)

Since cell death and cell proliferation are different processes, live cell counts were monitored over time in the four colon cancer cell lines, SW480, HCT-116, HCT-15, and HT-29 with 100 μM RRR-α- and RRR-γ-tocopherol treatment (Figure 2A–D). This was designed to assess the effects of the tocopherols on cell proliferation. In the HCT-116 and HT-29 (COX-2 positive) cell lines, RRR-α-tocopherol treatment resulted in a statistically significant decrease in cell proliferation, but the RRR-γ-tocopherol treatment resulted in a more pronounced decrease in cell proliferation compared with RRR-α-tocopherol. In fact, RRR-γ-tocopherol was statistically different from RRR-α-tocopherol in every cell line tested at 72 hours and in SW480, HCT-116, and HT-29 at 48 hours as demonstrated by post hoc analyses (Table 1). In the HCT-15 (COX-2 deficient) and SW480 (COX-2 deficient) cell lines, RRR-α-tocopherol treatment resulted in a statistically significant increase in cell growth compared with the vehicle, which was most evident at the 72-hour time interval. RRR-γ-tocopherol treatment resulted in a statistically significant reduction in proliferation with every cancer cell line tested. The HT-29 (COX-2 positive) cells demonstrated a slight resistance to RRR-γ-tocopherol treatment, initially (24-hour treatment) however, by 48 hours RRR-γ-tocopherol was as effective in HT-29 cells as in the other cells treated.

### Effects of RRR-α- and RRR-γ-tocopherol treatment on cell viability in human colon cancer cell lines compared with human normal colon cell line CCD-112CoN

The effects of 100 μM RRR-α- and RRR-γ-tocopherol treatments on cell viability were monitored with hemocytometer cell counts and the use of trypan blue dye following 72 hours in the normal nontransformed colon cell line CCD-112CoN (Figure 2E). RRR-γ-tocopherol treatment resulted in no cytotoxicity to normal colon cells, but demonstrated selectivity against cancer cell growth in all four colon cancer cell lines treated (Figure 2). RRR-α-tocopherol resulted in a statistically significant increase in CCD-112CoN cells.

### Effects of lower RRR-α- and RRR-γ-tocopherol treatment concentrations for extended time intervals on human colon cancer cell proliferation

**Figure 3:**
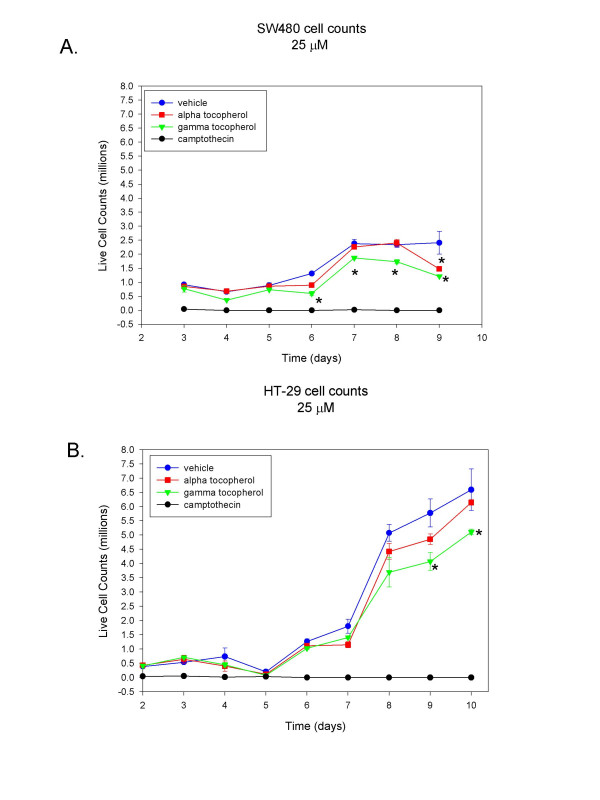
Live Cell Counts following a 25 μM α- and γ-tocopherol treatment at varying times up to ten days in SW480 cells (A) and HT-29 cells (B). (Data are representative of two independent trials performed in triplicate. *p < 0.05 vs. vehicle at corresponding concentration).

Since 100 μM is not a physiologically achievable concentration for dietary RRR-γ-tocopherol, we wanted to determine if similar results could be obtained with lower concentrations over longer time intervals. SW480, HCT-116, and HT-29 cells were treated with 25 μM of either RRR-γ-tocopherol or RRR-α-tocopherol for 9–10 days. Figure 3 shows the live cell counts as a function of time for the SW480 and HT-29 cell lines. RRR-γ-tocopherol treatment in the SW480 cells resulted in significantly less live cells compared to the vehicle treatment after 96 hours and time periods beyond 144 hours (p < 0.05). In the SW480 cells, RRR-γ-tocopherol-treated cell numbers were significantly less than the RRR-α-tocopherol treated up to 216 hours. In the HT-29 cell line, the RRR-γ-tocopherol treatment is statistically less at time periods longer then 216 hours. The effects of RRR-α-tocopherol, in the HT-29 cells, are not statistically different from that of vehicle-treated cells. The HCT-116 cells showed no statistical difference from the control for either RRR-α-tocopherol or RRR-γ-tocopherol treatment at any time point tested (data not shown).

### Effects of RRR-α- and RRR-γ-tocopherol treatment on apoptosis in human colon cancer cell lines

**Figure 4:**
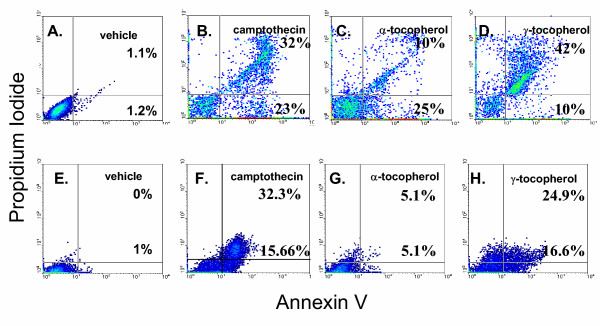
The annexin V/propidium iodide double staining assay following 100 μM tocopherol treatment in HCT-116 cells (Panels A-D) for 24 hours and SW480 (Panels E-H) cells for 70 hours. These data show RRR-γ-tocopherol is superior to RRR-α-tocopherol at inducing apoptosis. The percentages in right quadrants represent percentage apoptosis over the blank and are an average of at least two independent trials.

The effects of RRR-α-tocopherol and RRR-γ-tocopherol on apoptosis were measured by three independent assays on two selected colon cancer cell lines SW480 (COX-2 deficient) and HCT-116 (COX-2 inducible). In addition to the variable COX-2 expression, the SW480 and HCT-116 cell lines were selected for the apoptosis studies because these cell lines resulted in significant RRR-α-tocopherol-mediated cell death above the vehicle at 72 hours. It was reasoned that if RRR-α-tocopherol could mediate apoptosis; it would be seen in these two cell lines. First, annexin V/propidium iodide (PI) double staining was performed (Figure 4). This method identifies the percentage of cells that are in early (annexin V positive cells in lower right quadrant) and late apoptosis (annexin V and PI positive cells, double stained in the upper right quadrant). Both cell lines demonstrate significant apoptosis occurring following treatment with RRR-γ-tocopherol, but much less with the RRR-α-tocopherol treatment. In fact, the SW480 cells treated with RRR-α-tocopherol have no more than 11% of the cells undergoing early and late stage apoptosis combined. In both cell lines tested the ratio of RRR-γ-tocopherol-mediated late stage apoptosis is five times that of RRR-α-tocopherol.

**Figure 5:**
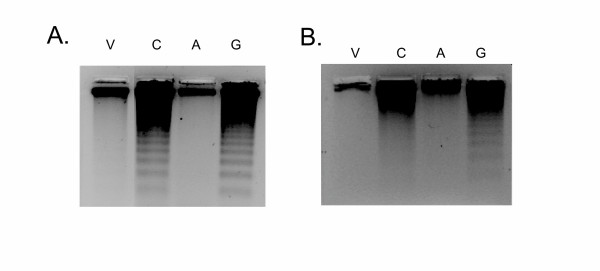
γ-Tocopherol treatment results in DNA laddering in HCT-116 cells (Panel A) and SW480 cells (Panel B) following a 100 μM tocopherol treatment for 48 hours (V = vehicle, C = camptothecin (+) control, A = alpha tocopherol, G = gamma tocopherol).

To confirm that apoptosis resulted from tocopherol treatment, a DNA laddering assay was performed (Figure 5). DNA laddering is only evident in the RRR-γ-tocopherol treated sample, but not the RRR-α-tocopherol treated sample in both SW480 and HCT-116 cell lines.

**Figure 6:**
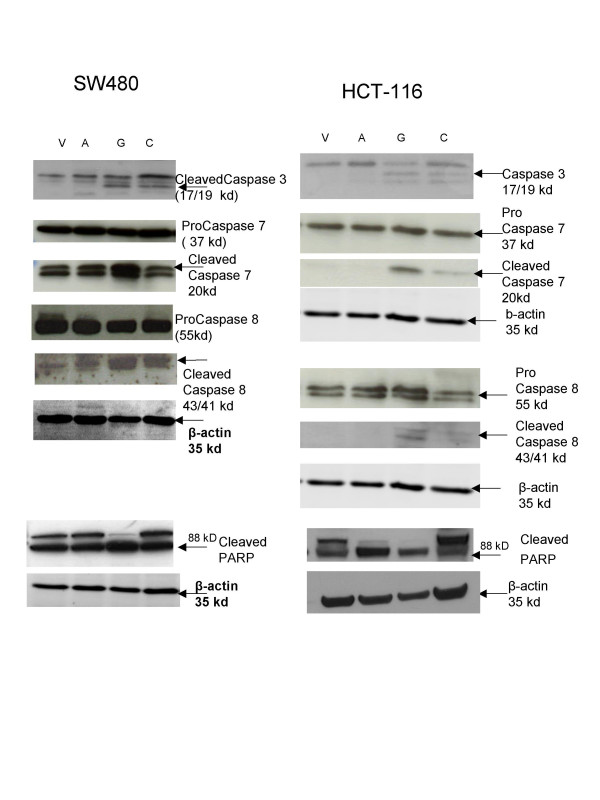
Western Blot analysis of SW480 (left) and HCT-116 (right) cell lysates following treatment with 100 μM tocopherols for 24 hours blotted with the antibodies to caspase 3, caspase 7, caspase 8, PARP, and β-actin as a loading control.

Further, we sought to determine which apoptotic pathway(s) are involved in the RRR-γ-tocopherol-mediated apoptosis, by monitoring caspase activation. Western blot analyses demonstrated that PARP, caspase 3, 7 and 8 are activated by cleavage in both cell lines with RRR-γ-tocopherol treatment, but not RRR-α-tocopherol treatment (Figure 6). Caspase 9 was not activated by cleavage in either cell line (data not shown).

### Intracellular accumulation of RRR-α- and RRR-γ-tocopherol treatment is both time and concentration dependent

**Figure 7:**
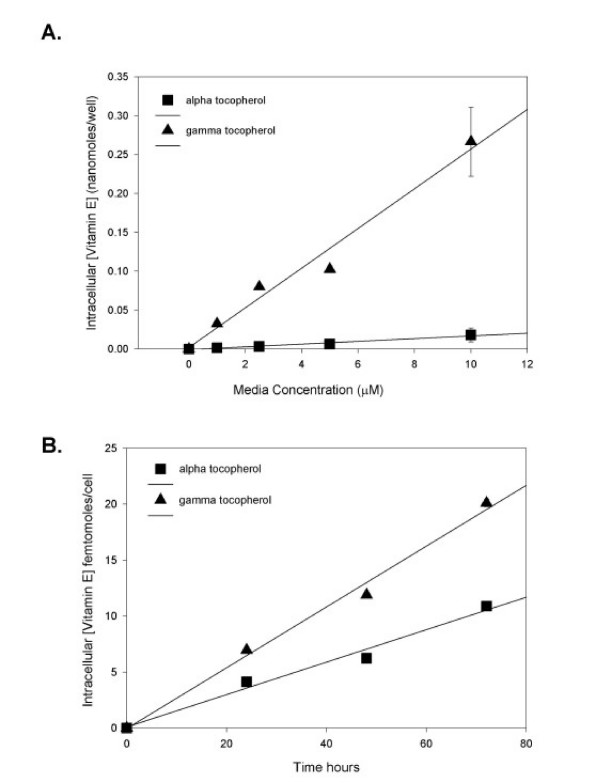
Cellular up take of RRR-α- and RRR-γ-tocopherol is time and concentration dependent, with RRR-γ-tocopherol accumulating to a higher intracellular concentration. A) SW480 cells were treated with varying concentrations of RRR-α- and RRR-γ-tocopherol for 24 hours. B) SW480 cells were treated with 100 μM RRR-α- or RRR-γ-tocopherol, collected, counted and subjected to HPLC analysis after 24, 48 and 72 hour treatments. Values are average of three independent experiments and error bars represent standard deviation. Time-dependent HPLC values were normalized to cell counts due to cell death at longer time intervals.

The intracellular accumulation of RRR-α- and RRR-γ-tocopherol in SW480 cells was determined as a function of the tocopherol concentration in the media (Figure 7A) and as a function of time (Figure 7B). Figure 7A demonstrates that the uptake of vitamin E is concentration dependent. We wanted to determine if the cellular up take during our treatment conditions (100 μM RRR-α- or RRR-γ-tocopherol at 24, 48 and 72 hours) followed a linear time dependent relationship. As shown in Figure 7B, the cellular uptake of RRR-α- and RRR-γ-tocopherol with respect to time is linear. The intracellular concentration of the vitamin E isoforms were normalized to the number of live cells in the sample at each time point. This was required since the tocopherols resulted in cell death at 100 μM. The intracellular concentrations of RRR-α- and RRR-γ-tocopherol were very low (femtomoles/cell) when compared to the amount of tocopherol added to the media (100 μM) but the RRR-γ-tocopherol intracellular uptake was significantly higher than that of RRR-α-tocopherol.

## Discussion

Recently published work has demonstrated disparity among the vitamin E isoforms, analogs, and metabolites to induce apoptosis. For example, VES and CEHC metabolites of vitamin E are strong apoptotic inducers, while α-tocopherol acetate (RRR- or all-rac- not specified), α-tocopheryl acetate (RRR- or all-rac- not specified) and α-tocopherol (RRR- or all-rac- not specified) are weak apoptotic inducers [17,18,20]. Gysin *et al*. used 25 μM RRR-γ-, RRR-α- and RRR-β-tocopherol, compared the proliferation effects, and determined that RRR-γ-tocopherol was best at inhibiting cell proliferation in human prostate carcinoma cells (DU-145 and LNCaP), human colorectal adenocarcinoma cells (CaCo2), and human osteosarcoma cells (SaOs-2). In this study, Gysin did not test the colon cancer cell line for apoptosis. They found no apparent cell death in the CaCo2 cells when using the trypan blue assay to test with 25 μM tocopherols. They further tested the DU-145 and LNCaP cell lines for apoptosis at the 25 μM RRR-γ-tocopherol concentration and found less than 3% of the cells were apoptotic. When employing 100 μM RRR-γ-tocopherol treatment to HCT-116 and SW480 cell lines we found significant apoptosis (Figures 4, 5 and 6). We suspect the reason for this discrepancy involves the cellular uptake of RRR-γ-tocopherol. We demonstrated that uptake of the tocopherols is concentration dependent. We suspect that the variation in apoptotic induction is due to the fact that we used a tocopherol concentration that was four times higher thereby significantly increasing cellular tocopherol uptake.

A number of studies have demonstrated that RRR-γ-tocopherol has chemopreventive properties not shared with RRR-α-tocopherol. For example, can lower levels of C-reactive protein [11], inhibit neoplastic transformation [12], suppress ras p-21 [10], inhibit COX-2 activity [13], down regulate cyclins [14], and up regulate PPAR γ [22]. Our study is the first to demonstrate that RRR-γ-tocopherol can result in cell death and apoptosis to colon cancer cell lines, with no significant cytotoxic effects to normal colon cells.

We acknowledge that 100 μM concentrations of tocopherols used in cell culture experiments is not an achievable plasma level through oral administration in humans but could be achieved by other methods such as intravenous vitamin E-liposomes [24]. The intravenous use of antioxidant liposomes could by-pass the bioselective processes of the liver and rapidly increase plasma and tissue levels of RRR-γ-tocopherol beyond what can be achieved by oral administration. In vivo experiments in a rat model suggest that colonocytes receive RRR-γ-tocopherol from both plasma and the contents of the digestive tract [10]. In humans, consuming a Western diet the dietary levels of RRR-γ-tocopherol are much higher than that of RRR-α-tocopherol. It would be expected, therefore, that RRR-γ-tocopherol would make a significant contribution to the total tocopherol content of colonocytes. Levels of RRR-γ-tocopherol in human colonocytes are indeed higher than that of RRR-α-tocopherol [25]. These data suggest that levels of RRR-γ-tocopherol in colonocytes could be much higher than reflected by plasma levels where ratio of RRR-γ-tocopherol to RRR-α-tocopherol is about 1 to 10 [10,25].

Our HPLC results also indicate that at the 100 μM vitamin E concentrations used to treat these colon cancer cells only femtomoles of vitamin E are available inside the cell, yet, at these intracellular concentrations (femtomoles/cell), cell death and apoptosis were induced. It is a significant finding that intracellular concentrations in the femtomole range can induce cell death in colon cancer cells. To date, it is not known whether these intracellular concentrations of femtomoles can be achieved in vivo through dietary means. Further research needs to be performed to determine what physiological intracellular and tissue concentrations of vitamin E isoforms are achievable within various tissues. In addition, our HPLC results indicate that the longer the cells are exposed to vitamin E isoforms, the higher the intracellular accumulation of vitamin E. With this in mind, we demonstrated that colon cancer cell lines exposed to physiological concentrations of RRR-γ-tocopherol for extended periods of time undergo reduced cell proliferation. These data suggest that dietary RRR-γ-tocopherol could have a protective effect over a long time span through a gradual tissue accumulation reaching the femtomole/cell range and thereby be chemopreventive.

We can only speculate at this time on the molecular basis for the differences in the growth inhibitory effects on the different colon cancer cell lines. There are, however, some interesting data correlations that can be made between the molecular characteristics of the cell lines and the effects of the tocopherols in this study. For example, HCT-15 and SW480 cells are COX-2 negative, HCT-116 cells are COX-2 inducible and HT-29 cells over express COX-2. The growth inhibitory effect of RRR-α-tocopherol is more pronounced in cells that over express COX-2 (HT-29 cells). Preliminary work in our laboratories indicate that tocopherols suppress COX-2 in HT-29 and CaCo2 cells (unpublished) and it is possible that some of the apoptotic effects of tocopherols are mediated by COX-2 suppression and hence are more apparent in cells that express COX-2. The HT-29 cells are slightly more resistant to cell death than the other cell lines as demonstrated by the increase in cell number over the vehicle at 24 hours. This may be due to the COX-2 expression in HT-29 cells. It is known that the expression of COX-2 can mediate apoptotic resistance in cancer cells [26]. Further investigation is underway in our laboratories to explore if the effects of RRR-α- and RRR-γ-tocopherol on COX-2 expression and apoptosis in these cell lines. Jiang *et al. *have demonstrated that RRR-γ-tocopherol, but not RRR-α-tocopherol decreases the activity of COX-2 in IL-1 and LPS-stimulated macrophages. Jiang *et al. *also demonstrated that, in macrophages, neither COX-2 protein or mRNA expression was affected by tocopherol treatment, leading to the speculation that RRR-γ-tocopherol may compete for the arachidonic acid binding site which leads to protein inhibition and reduced activity [13]. Additionally, SW480 and HT-29 cells have a truncated APC gene, while the HCT-116 cells have a wild type APC gene. The APC protein is involved in the regulation of β-catenin. In cancer, mutations in the APC gene allow for accumulation of β-catenin. β-Catenin is a nuclear transcription factor that modulates genes associated with cell proliferation. Accumulation of β-catenin protein leads to enhanced cell proliferation by dysregulation of the wnt pathway [27]. Our data show that HCT-116 cells are not affected by 25 μM tocopherol treatment at any time point tested (maximum 10 days), while the SW480 and HT-29 cell lines show a reduction in cell proliferation, indicating that RRR-γ-tocopherol may have an effect on cell proliferation by regulation of the wnt pathway. It is well documented that disruption of the wnt pathway occurs early in the carcinogenesis process. The potential modulation of the wnt pathway by RRR-γ-tocopherol needs to be investigated further.

Jiang *et al. *has performed mechanistic studies suggesting that RRR-γ-tocopherol induces prostate cancer cell death by disrupting the *de novo *sphingolipid pathway which is important for the biosynthesis of ceramide [28]. Ceramide has received considerable attention as a potential second messenger important for inducing apoptosis [29-31]. In the sphingolipid study, vitamin E did not show an apoptotic effect on LNCaP prostate cells until 72 hours following treatment. The apoptotic event correlated with the increase and accumulation of dihydroceramide and dihydrosphingosine. Our data demonstrates a similar time differential before apoptosis occurs in the colon cancer cell lines we tested. It is possible that RRR-γ-tocopherol may interfere with the *de novo *sphingolipid pathway in the colon cells as well as prostate cells to induce apoptosis by production of ceramide. There are different compartmentalized pathways of ceramide signaling, each having unique molecular signatures for apoptosis/proapoptosis [32]. For example, lysosomal ceramide generation results in Cathespin D and BID-mediated activation of caspase 9 and 3. The generation of ceramide in the ER or mitochondria will cause protein phosphatase 1-mediated activation of caspase 9. Ceramide generation in the lipid membrane can affect signaling pathways generated by receptors such as Fas resulting in the activation of caspase 8. Our data differs from that of Jiang *et al. *with respect to caspase 9 cleavage. We did not observe caspase 9 cleavage in the colon cells whereas Jiang et al. observed caspase 9 cleavage in the prostate cells. This means that if vitamin E is following a pathway involving ceramide biosynthesis, the cellular sublocalization of vitamin E mediation of ceramide is different in colon from that of the prostate gland. It is likely that if RRR-γ-tocopherol interacts to interfere with sphingolipid synthesis in the colon it happens in the plasma membrane as we have detected cleavage of caspase 8. This pathway requires further investigation in the colon.

Our research group previously found that RRR-γ-tocopherol up regulates the expression of peroxisome proliferator activator receptor-gamma (PPAR-γ) more effectively than RRR-α-tocopherol [22]. PPAR γ is a key molecular target for cancer chemoprevention. Many *in vitro *and xenograft studies have demonstrated that PPAR γ ligands are anti-tumorigenic due to anti-proliferative, pro-differentiation and anti-angiogenic effects [33-38]. RRR-γ-tocopherol mediated cell death and apoptosis may follow a PPAR γ dependent mechanism in HCT-116, SW480 and HT-29 cell lines, but not in HCT-15 cells which have a point mutation (K422Q) and express a mutant form of PPAR γ. This mutation makes them resistant to ligand activation. Our data (Figure 2) shows that RRR-γ-tocopherol induces cell death in the HCT-15 cells, therefore we suspect that the RRR-γ-tocopherol mediated cell death does not occur through a PPAR γ dependent mechanism only but that other pathways are involved. It is possible that vitamin E may activate multiple pathways in concert resulting in apoptosis and cell death of colon cancer cells of different prosurvival factors result in the variations among cell lines to induce apoptosis (as in the 24-hour data obtained with the HT-29 cell line). The potential pathways of tocopherol activity under investigation in our laboratories include mechanisms related to lipid metabolism (COX-2, lipoxygenase, and sphingolipid pathways) and to pathways not related to lipid metabolism (wnt pathway).

## Conclusion

The effectiveness of γ-tocopherol to inhibit cell proliferation in these human colon cell lines vary according the individual molecular characteristics of each line. This study indicates that RRR-γ-tocopherol mediated apoptosis in colon cells largely follows a death receptor pathway since caspase 8, but not caspase 9 cleavage was detected. This study demonstrates that both physiological (25 μM) and pharmacological concentrations (100 μM) of RRR-γ-tocopherol effectively reduced cell proliferation in malignant colon cancer cell lines that possess different molecular signatures without damage to normal colon cells. Based on our studies, it is possible that both physiological and pharmacological concentrations of RRR-γ-tocopherol may have a role in the prevention and therapy of colorectal cancer. Physiological concentrations of RRR-γ-tocopherol as taken in the diet may be chemopreventive, while pharmacological concentrations of RRR-γ-tocopherol may be used in conjunction with chemotherapeutic agents as adjunctive therapy enabling the reduction in the concentrations of toxic chemotherapeutic agents, maintaining the levels of cell cancer cell death while reducing the death of normal colon cells.

## Competing interests

Our laboratories have no financial interest, arrangement or affiliation with any product or organization that could be perceived as a real or apparent conflict of interest in the context of this manuscript.

## Authors' contributions

SC conceived the study, and participated in the study design, performed data analysis, aided in troubleshooting assays and drafted the manuscript. WS participated in the design and coordination of the delivery of the vitamin E isoforms to the cells and the HPLC analysis and manuscript preparation. SW, DS, DM and PG carried out the cell proliferation and apoptosis assays, while QM, SL and HY performed the western blot analyses. KK conceived the study along side SC, participated in the study design and guided trouble shooting assays, and manuscript preparation. All authors read and approved the final manuscript.

## Pre-publication history

The pre-publication history for this paper can be accessed here:



## Figures and Tables

**Table 1:** Statistical results comparing the trypan blue live-cell means of the tococpherol-treated sampleswith vehicle-treated sample at 48 and 72 hours following 100 μM tocopherol treatment

	SW480		HCT-15		HCT-116		HT-29		CCD-112CoN
Treatment Agent	p-value 48 hour	p-value 72 hour	p-value 48 hour	p-value 72 hour	p-value 48 hour	p-value 72 hour	p-value 48 hour	p-value 72 hour	p-value 72 hour

alpha tocopherol	0.034	<0.001	0.775	<0.0001	0.996	0.084	0.000	<0.0001	0.020
gamma tocopherol	0.0001	<0.001	0.112	<0.0001	0.0005	0.005	<0.0001	<0.0001	0.927
15-deoxy Δ12, 14 PGJ_2_	0.0004	<0.001	N/A	N/A	N/A	N/A	N/A	N/A	N/A
troglitazone	N/A	N/A	N/A	N/A	0.0003	0.0005	0.0000	<0.0001	<0.0001
camptothecin	N/A	N/A	0.853	<0.001	N/A	N/A	N/A	N/A	N/A
alpha vs. gamma	<0.018	<0.001	0.416	<0.001	0.0005	0.001	0.0000	<0.0001	0.0117

*Comparison of means was performed using one-way anova and a Tukey's post hoc analysis. Data are representative of three independent trials performed in triplicate.
